# Zonulin family peptide is not a predictive marker for development of minor postoperative complications: an observational study in patients after colorectal surgery

**DOI:** 10.3389/fmed.2025.1605142

**Published:** 2025-08-01

**Authors:** Agnes Knott, Alexander Müller, Gabriele Paul, Lampros Kousoulas, Roman Huber, Ann-Kathrin Lederer

**Affiliations:** ^1^Center for Complementary Medicine, Department of Medicine II, Faculty of Medicine, Medical Center – University of Freiburg, Freiburg, Germany; ^2^Department for General and Visceral Surgery, Faculty of Medicine, Medical Center – University of Freiburg, Freiburg, Germany; ^3^Department of General, Visceral and Transplant Surgery, University Medical Center of the Johannes Gutenberg University, Mainz, Germany

**Keywords:** visceral surgery, anastomotic leakage, wound infection, outcome, biomarkers, prevention and control, perioperative medicine

## Abstract

**Background:**

Postoperative complications after colorectal surgery are still challenging. Zonulin family peptide (ZFP) has been discussed as a potential marker of intestinal permeability and postoperative complications. The aim of this trial was to investigate whether ZFP allows early diagnosis of postoperative complications after colorectal surgery.

**Method:**

We performed a monocentric, observational study among patients undergoing elective colorectal surgery between April 2018 and April 2019. Patients of all ages regardless of sex undergoing colorectal surgery without creation of ostomy were eligible for inclusion. Emergency surgery were not considered. Blood samples were taken preoperatively as well as on the 3rd, 6th and 9th postoperative day. All postoperative complications were classified according to Clavien-Dindo.

**Results:**

Overall, 67 patients participated in the trial, 51% were male, average age was 58 years. Thirty-seven patients (55%) developed postoperative complications, mostly mild or moderate (Clavien-Dindo I and II). Almost all patients had a ZFP concentration in serum above the normal reference range preoperatively. Postoperatively, there was a significant decrease of ZFP on the 3rd postoperative day compared to the preoperative concentration of ZFP, but there was no association between ZFP levels and development of postoperative complications.

**Conclusion:**

The results suggest that ZFP is not able to predict minor postoperative complications after colorectal surgery.

## Introduction

Postoperative complications after colorectal surgery remain an enormous burden, contributing to high morbidity and mortality rates among affected patients and additional costs to healthcare systems (1). The most common postoperative complications include postoperative ileus (20%), surgical site infections [including superficial wound infections (30%) and abscesses (12%)], bleeding (4%) and the development of an anastomotic leakage (AL) (23%) (2). In healthy individuals, a well-working intestinal mucosal barrier prevents bacterial translocation. Every surgical abdominal manipulation, even if it is planned, leads to a disruption of this sensitive equilibrium. Bacterial translocation has been shown to occur after major abdominal surgery and more often in patients with postoperative infectious complications (3, 4). Gut failure is common in critical ill patients and is strongly suggested to be a main contributor to multiple organ failure (5). The dysfunction of intestinal barrier function leads to a vicious circle; the increased exposure to translocated antigens can provoke an extensive, even life-threatening immune reaction, which in turn aggravates the critical situation (6). Early diagnosis of postoperative complications is crucial to prevent subsequent complications. Although biomarkers for the early detection of intestinal mucosal barrier dysfunction in surgical patients would be highly desirable, current data are still inconsistent. One molecule, which is discussed to picture the gut barrier function, is zonulin. Zonulin is a gut barrier-regulating protein representing a precursor of haptoglobin2, and being secreted by the intestinal membrane, for example after contact with bacteria (7, 8). The main effect of zonulin is the opening of intercellular tight junctions, which increases intestinal permeability, thus allowing exchange between intestinal cells (7, 9). This function ensures the exchange between intestinal cells. Zonulin has been measured over the last years using a standard enzyme-linked immunosorbent assay (ELISA). In recent years, it has been shown that zonulin ELISA not only measures zonulin but also a variety of zonulin-related peptides such as properdin, which is why the term “zonulin family peptide” (ZFP) is now used (10, 11). The role of ZFP in regulating gastrointestinal permeability remains a subject of debate (12). Like zonulin, ZFP translocates into the bloodstream, enabling its detection in serum. It is still unknown how colorectal resection affects ZFP secretion. We hypothesized that surgery may lead to hypersecretion of ZFP and might be associated with postoperative complications due to the fostering of bacterial translocation. Therefore, the aim of this study was to evaluate whether ZFP could serve as a suitable systemic biomarker for the early diagnosis of postoperative complications.

## Materials and methods

We performed a prospective, monocentric observational study at the Department for General and Visceral Surgery, University Medical Center of Freiburg between April 2018 and April 2019. Eligible for inclusion were all patients of full age, who underwent elective colorectal surgery without creation of ostomy (e.g., hemicolectomies or sigmoid resections). Exclusion criteria were intake of probiotics at least 4 weeks before study as well as regular intake of opiates, bowel stimulants or antiemetics. Patients who underwent emergency surgery or with a pre-existing ostomy were not considered. Patients who canceled their surgery or whose surgery was postponed were excluded from the study. Written informed consent was obtained from all patients prior to the study inclusion. Before onset, the study was approved by the local ethics committee (EK-Freiburg: 535/17) and registered at the German Clinical Trials Register (DRKS00014059).

### Sample collection

The study followed a structured timeline to collect clinical data and blood samples. All patients were seen preoperatively, and were thoroughly informed about the study. After signing the written informed consent, baseline characteristics, including demographic data and comorbidities were obtained. Following, patients were visited on the postoperative days 3, 6 and 9 to document postoperative recovery and occurrence of postoperative complications. Data was obtained by a personal interview or were taken from the electronic patient file as well as from surgical or anesthesiologic protocols. For ZFP analysis, blood samples were collected preoperatively and on postoperative days 3, 6, and 9 using standardized serum tubes commonly employed for routine blood collection. Patients received a single intraoperative antibiotic prophylaxis with 2,000 mg cefazolin and 500 mg metronidazole. All patients were treated according to a standardized protocol for early recovery: minimally invasive surgical technique, if possible without insertion of drains, epidural analgesia, rapid transition to oral pain management, early mobilization, early resumption of oral nutrition with easily digestible fluids and light meals within 24 h postoperatively. Postoperative complications were classified using the Clavien-Dindo classification system (13). Any deviation from the expected postoperative course was classified as a complication. Patients who experienced any postoperative complication classified as Clavien-Dindo classification I or higher were assigned to Group A. Patients without any complications (Clavien-Dindo classification 0) were assigned to Group B. Minor complications were defined as grade I and II according to Clavien-Dindo classification, while major complications were classified as grade III or higher. Major complications included AL requiring reoperation or intervention (grade III or higher). Additionally, clinical parameters such as fever (≥ 38.5°C), delayed return to normal oral intake, persistent nausea, and vomiting were documented.

### Processing of samples

Blood samples were processed immediately. All samples were centrifuged at 3000 rpm for five minutes. Serum aliquots were stored at −80°C until analysis. To ensure the stability of the samples, the samples were analyzed after 3 months at the latest. ZFP levels were measured using a validated ELISA (IDK^®^ Zonulin (Serum) ELISA, Immundiagnostik AG, Germany) performed at a specialized diagnostic laboratory (IMD Institut für Medizinische Diagnostik Berlin-Potsdam GbR, Berlin, Germany). All measurements were conducted in duplicate, and average values were used for analysis. The assay’s normal reference range for healthy individuals was < 38 ng/ml.

### Statistics

The primary aim of this study was to evaluate whether ZFP is a suitable biomarker for the early diagnosis of postoperative complications. For this purpose, the ZFP levels of patients with and without complications were compared. The sample size calculation was based on an assumed large effect size (Cohen’s d = 0.8), a significance level of 5%, and a power of 80%, resulting in a required total sample size of 52 patients (26 patients with complications vs. 26 patients without complications). A 1:1 group ratio was assumed to be realistic, as current literature indicates that approximately 50% of patients develop postoperative complications after colorectal surgery (14). During the study, it became evident that the initially planned drop-out rate of 20% (12 patients) was too low. In order to reach the planned sample size, the recruitment was continued after 64 patients. All data was entered in a predefined table without knowing whether patients had complications or not. The occurrence of complications was added after the database was closed. For comparison, all patients were then divided into two groups (group A: with complications, group B: without complications). All results were tested for normal distribution by Kolmogorov-Smirnov test. Continuous data were reported as mean ± standard deviation, while categorical data are presented as absolute value and percentage of all corresponding patients. Comparisons between patients with and without complications were made using Student’s *t*-test or Mann-Whitney U test for continuous variables and chi-square or Fisher’s exact test for categorical variables. A paired *t*-test was used to compare the results of different days in one patient. The correlations between ZFP levels and BMI or age were assessed using Spearman’s rank correlation coefficient. Multivariate logistic regression analysis was performed to evaluate the independent predictive value of preoperative ZFP levels for postoperative complications, adjusting for potential confounders such as age, BMI, and comorbidities. Statistical significance was defined as *p* < 0.05.

## Results

**FIGURE 1:**
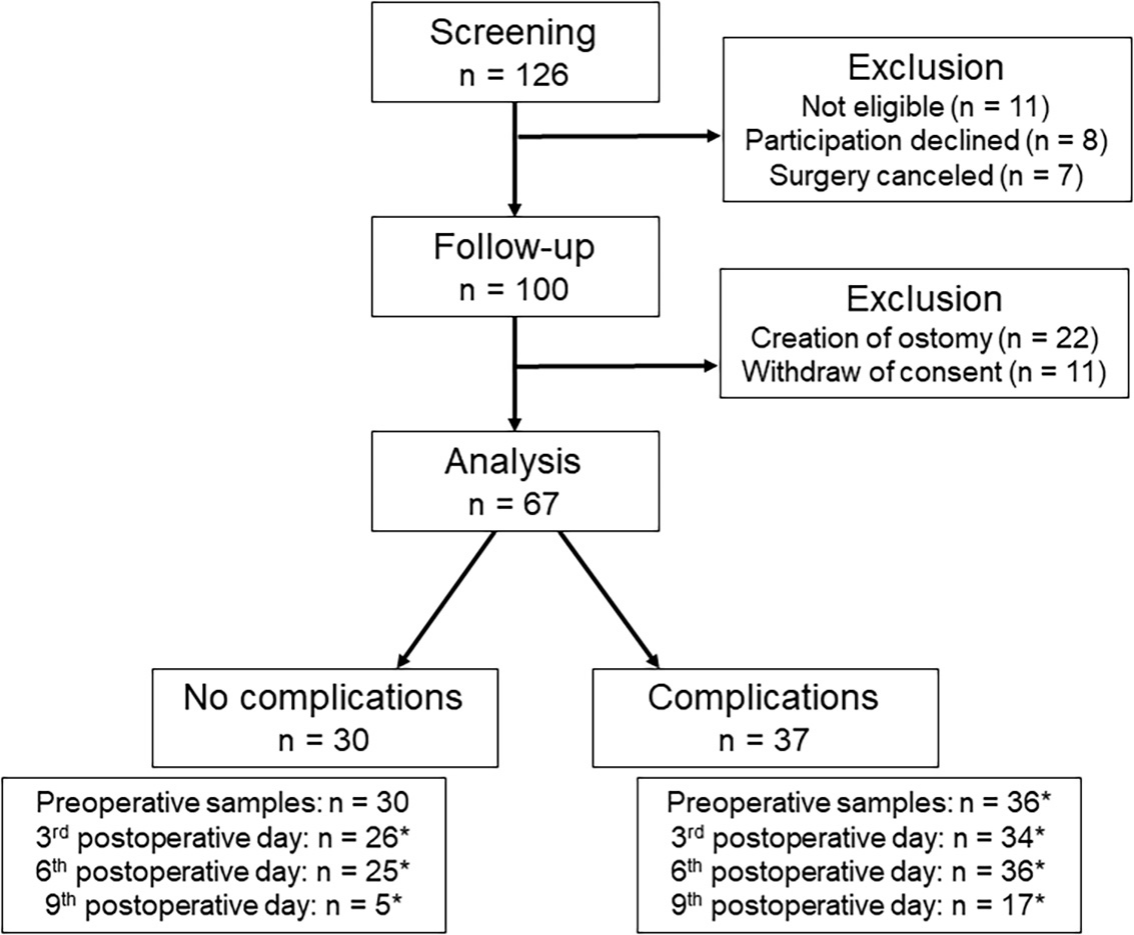
Process of screening and inclusion. *Not all patients could be analyzed to all times, because patients refused to blood sampling (*n* = 18), the measurement was not possible (*n* = 1), or the patients had already been discharged (*n* = 40).

A total of 100 patients were included in the study after screening, but only 67 of them could be analyzed. In 22 patients, an unplanned ostomy had to be created during surgery leading to their exclusion after surgery. Further 11 patients were no longer interested in participating after surgery. An overview of the screening and inclusion process is shown in Figure 1.

Slightly more than half of the patients (51%, *n* = 34) were male. Patients were on average 58.0 (±15.9) years old. Descriptive patients’ data is shown in Table 1. The majority of operations were performed due to cancer (42%, *n* = 28). Further indications included the restoration of bowel continuity (19%, *n* = 13), for example after a previous Hartmann’s procedure. Overall, 55% of the patients (*n* = 37) experienced postoperative complications. Most of the complications were minor complications (40%, *n* = 27). Patients in group A (with complications) were significantly older than patients in group B (without complications, 61.7 vs. 53.3 years, *p* = 0.016), and had a significantly longer hospital stay compared with group B (10.6 vs. 8.5 days, *p* = 0.001). All types of complications and further clinical data are shown in Table 2.

**TABLE 1:** Descriptive data of all included patients and comparison of subgroups.

	Total (*n* = 67)	A (*n* = 37)	B (*n* = 30)	P°
Age [years ± SD]	58.0 ± 15.9	61.7 ± 15.2	53.3 ± 15.7	**0.016**
Sex [% (*n*)]				
- Male	51% (34)	54% (20)	47% (14)	0.361
- Female	49% (33)	46% (17)	53% (16)	
BMI [kg/m^2^ ± SD]	25.1 ± 4.8	25.1 ± 4.4	25.2 ± 5.4	0.909
Pre-existing illnesses [% (*n*)]				
- Cardiovascular disease*	40% (27)	46% (17)	33% (10)	0.213
- Diabetes mellitus	6% (4)	10% (3)	3% (1)	0.390
- Chronic renal failure	3% (2)	3% (1)	3% (1)	0.699
- Inflammatory bowel disease	13% (9)	8% (3)	20% (6)	0.145
Nicotine abuse [% (*n*)]				
- Currently	21% (14)	19% (7)	23% (7)	0.544
- Formerly	28% (19)	24% (9)	33% (10)	
Alcohol abuse [% (*n*)]				
- Regularly (> 20 g/day)	8% (5)	14% (5)	0% (0)	**0.017**
- Occasionally (< 20 g/day)	60% (40)	46% (17)	77% (23)	
Cancer [% (*n*)]	42% (28)	51% (19)	30% (9)	0.065
First bowel movement (days ± SD)	2.6 ± 0.6	3.3 ± 1.5	3.0 ± 1.2	0.432
LOHS [days ± SD]	9.7 ± 3.1	10.6 ± 3.1	8.5 ± 2.73	**0.001**
Laparoscopic surgery [% (*n*)]	57% (38)	59% (22)	53% (16)	**0.013**
Laparotomic surgery [% (*n*)]	43% (29)	57% (21)	27% (8)	
Type of surgery [% (*n*)]				
- Left hemicolectomy	6% (4)	8% (3)	3% (1)	0.442
- Right hemicolectomy	36% (24)	43% (16)	27% (8)	
- Sigma resection	31% (21)	24% (9)	40% (12)	
- Restoration of continuity	19% (13)	19% (7)	20% (6)	
- Ileocecal resection	5% (3)	3% (1)	7% (2)	
- Other^+^	3% (2)	3% (1)	3% (1)	

The bold values indicate the significant values. Percentage of all patients in the corresponding group. *Including atherosclerosis, hypertension, stroke and rhythm disorders. ^+^Transverse resection and proctocolectomy. °Fisher’s exact text or Mann-Whitney-U-test, depending on the type of variable. LOHS, length of hospital stay; SD, standard deviation; A, with postoperative complication; B, without complications.

**TABLE 2:** Overview of clinical data and postoperative complications.

Clavien-Dindo classification	% (*n*)		
Grade 0	45% (30)		
Grade 1	15% (10)		
Grade 2	25% (17)		
Grade 3	12% (8)		
- 3a	6% (4)		
- 3b	6% (4)		
Grade 4	3% (2)		
- 4a	3% (2)		
- 4b	0% (0)		
Grade 5	0% (0)		
**Minor complications (Grade I and II)**	40% (27)		
**Major complications (Grade III–V)**	15% (10)		
**Acute renal failure**	3% (2)		
**Anastomotic leakage**	0% (0)		
**Paralytic ileus**	22% (15)		
**Pneumonia**	3% (2)		
**Revision surgery**	6% (4)		
**Urinary tract infection**	12% (8)		
**Wound infection**	2% (1)		
	**3. POD**	**6. POD**	**9. POD**
**Nausea [yes A/B% (*n* A/B)]**	62/55% (23/16)	41/40% (13/8)	24/0% (4/0)
**Extent of nausea [0–10 ± SD]**	3.6 ± 3.5	2.3 ± 3.3	0.7 ± 1.5
**Vomiting [yes A/B% (*n* A/B)]**			
- Once	19/24% (7/7)	18/5% (6/1)	15/0% (3/0)
- More than once	46/10% (17/3)	12/10% (4/2)	0/0% (0/0)
**Fever > 38.5°C [yes A/B% (*n* A/B)]**	5/7% (2/2)	0/0% (0/0)	0/0% (0/0)

Percentage of all patients, who were able to give response. POD, postoperative day; SD, standard deviation; A, with postoperative complication; B, without complications.

On the 3rd postoperative day, more than half of the patients suffered from nausea (59%, *n* = 39, which improved only slightly by postoperative day 6 (40%, *n* = 21). The majority of patients also had to vomit on the 3rd postoperative day (51%, *n* = 34), some of them several times (30%, *n* = 20). Fever was a rare symptom, which only occurred in 4 patients (6%) on the 3rd postoperative day. The occurrence of fever or nausea and the extent of nausea did not differ significantly between the patients with and without complications. Patients who experienced repeated vomiting on the 3rd postoperative day were significantly more likely to belong to the complication group (Group A). This can be easily explained, for example, by the need for nasogastric tube insertion. In 17 patients (85%) with repeated vomiting, further treatment was required.

### Zonulin family peptide (ZFP) levels

Postoperatively, it was possible to analyze a total of 209 blood samples (78% of all planned blood samples, Figure 1). Due to discharges, only 22 patients (33% of all patients) could be analyzed on postoperative day 9. Corresponding blood samples for all days were available in 20 patients (30% of all patients). Corresponding samples from preoperative to postoperative day 3 and postoperative day 6 were available in 53 patients (79% of all patients). Corresponding samples from preoperative to postoperative day 3 were available in 59 patients (88% of all patients). Before surgery, ZFP was on average 48.9 ± 10.6 ng/ml, and was thus above the normal reference range (< 38 ng/ml) in 82% patients (*n* = 55). Postoperatively, ZFP decreased initially to 42.4 ± 8.8 ng/ml and then increased slightly to 46.3 ± 9.6 ng/ml. There was no significant difference in ZFP levels between patients with and without complications. All ZFP results are shown in Table 3.

**TABLE 3:** Levels of zonulin family peptide (ZFP, ng/ml) before and after surgery and percentage of patients, who had ZFP levels above the normal reference range (< 38 ng/ml).

Absolute	[ng/ml] ± SD	A	B	P*
**Preoperative**	48.9 ± 10.6	49.2 ± 9.6	48.7 ± 12.0	0.854
**3. POD**	42.4 ± 8.8	40.8 ± 6.8	44.5 ± 10.7	0.112
**6. POD**	46.3 ± 9.6	45.7 ± 8.8	47.1 ± 10.8	0.569
**9. POD**	45.5 ± 11.9	45.4 ± 13.0	45.5 ± 8.4	0.998
**Above range**	**Total (*n* = 67)**	**A (*n* = 37)**	**B (*n* = 30)**	**P°**
**Preoperative**	82% (55)	87% (32)	77% (23)	0.235
**3. POD**	67% (40)	65% (22)	69% (18)	0.465
**6. POD**	80% (40)	78% (28)	84% (21)	0.397
**9. POD**	73% (16)	71% (12)	80% (4)	0.581

Percentage of all patients, who were able to give response. *t-test comparing A and B; °Fisher’s exact text comparing A and B. POD, postoperative day; SD, standard deviation; A, with postoperative complication; B, without complications.

**FIGURE 2:**
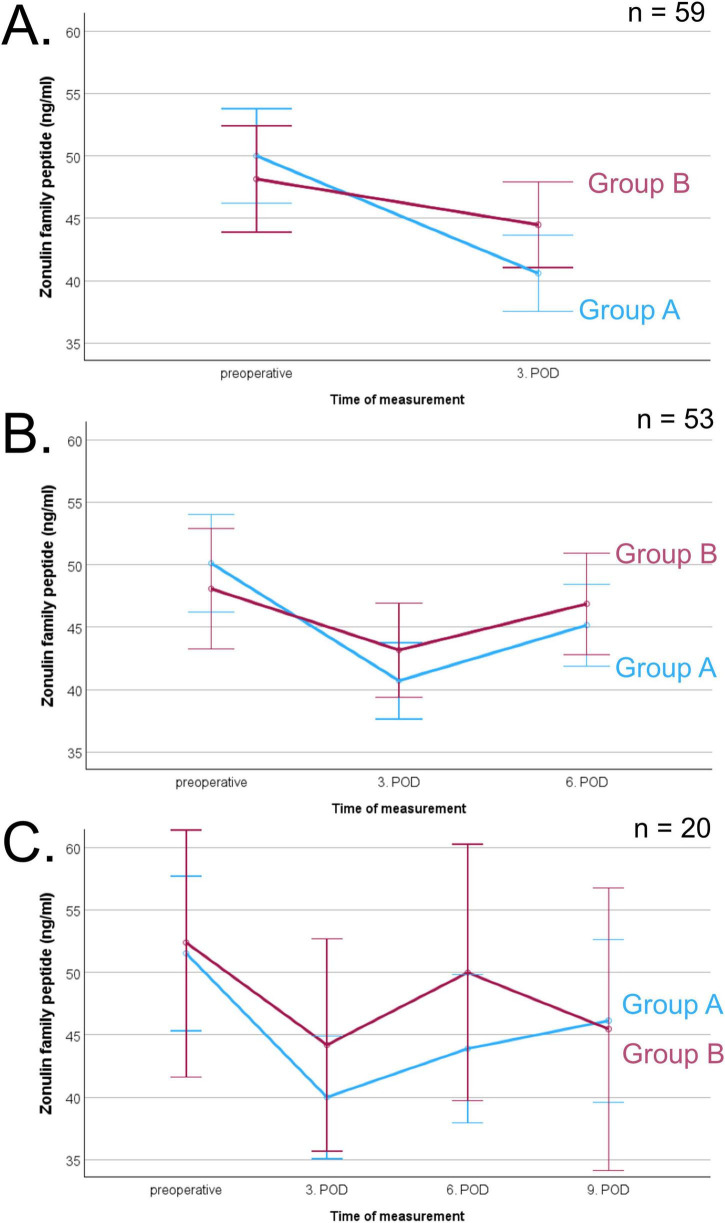
Course of zonulin family peptide (ZFP, pg/ml) before and after surgery of corresponding blood samples, separated in patients with (group A, blue line) and without complications (group B, red line), error bars represent 95% confidence intervals. **(A)** Comparison of preoperative and 3rd postoperative day (POD) measurement in 59 patients indicating an effect of time (*p* < 0.001), **(B)** Comparison of preoperative, 3rd and 6th POD measurement in 53 patients indicating an effect of time (*p* < 0.001), **(C)** Comparison of all times points in 20 patients indicating an effect of time (*p* = 0.007).

The course of ZFP levels is shown in Figure 2. Due to varying availability of blood samples on different postoperative days, the analyses were conducted separately for different days. Repeated measures ANOVA did not reveal a significant difference between group A and group B at any time point. There was a significant main effect of time on ZFP levels (Figure 2A: *p* < 0.001; Figure 2B: *p* < 0.001; Figure 2C: *p* = 0.007), indicating changes in ZFP levels irrespective of group affiliation after surgery. Postoperatively, there was a significant decrease in ZFP (preoperative to 3rd postoperative day *p* < 0.001, preoperative to 6th postoperative day *p* = 0.011, preoperative to 9th postoperative day *p* = 0.003). There was a significant increase on ZFP between postoperative days 3 and 6 (*p* < 0.001).

No significant correlation was observed between ZFP levels and age or BMI. ZFP levels did not differ significantly between patients diagnosed with cancer and those without malignancies. Likewise, no significant differences in ZFP levels were observed based on patients’ comorbidities.

## Discussion and conclusion

The purpose of this study was to explore the potential role of ZFP as a biomarker for the early detection of postoperative complications following colorectal surgery. Interestingly, the findings suggest that ZFP is not a reliable biomarker for identifying patients with postoperative complications, particularly of minor ones. The study provides results on the biodynamics of ZFP before and after colorectal resection. One of the study’s strengths is that ZFP levels were measured at multiple pre- and postoperative time points in a structured manner with strictly standardized procedures for preanalytics (see methods). However, it should be noted that no measurements were taken on postoperative days 1 and 2, which represents a limitation, particularly in light of the presumed short half-life of ZFP. Clear in- and exclusion criteria strengthen the study by ensuring a quite homogeneous patient group. The small sample size, resulting from the strict selection of patients, is, however, a limitation of the study. The main risk factor for complications was, as expected, age. The fact that younger patients experience fewer complications after colorectal surgery has also been demonstrated in other studies (15, 16). In our study, we documented the complications very carefully. Even small deviations from the normal course were regarded as complications. We have been able to reach the initial planned sample-size in both groups. The rate of postoperative ileus in our cohort appears high at 22%, but is consistent with the findings of others studies, which report that around 20% of patients are unable to tolerate oral food intake in the first few days after surgery (17). In fact, there are also studies in which almost one in two patients suffered from postoperative ileus after colorectal resection (18). It was particularly interesting that one of the most significant postoperative complications after colorectal resection, AL, did not occur in our cohort. According to the literature, AL affects up to 20% of patients after colorectal resection, so we had actually anticipated its occurrence in our cohort. One possible reason for this could be that 22 patients underwent an unplanned, primarily protective stoma creation during surgery. The operating surgeon must have therefore assumed a high risk of AL based on the intraoperative findings. Factors that may have contributed to the intraoperative decision to create an ostomy include the quality and perfusion of the anastomosis, operative time, the surgeon’s experience, and blood loss (19). Overall, it must be acknowledged that we cannot determine whether ZFP play a role in AL, as no AL was diagnosed in our study. We predominantly observed minor complications in this study, such as postoperative gastrointestinal paralysis.

Initially, we planned to measure zonulin. During the study, the ELISA test for measuring zonulin proved to be problematic, as several publications suggested that it does not measure zonulin but rather ZFP (10, 11). Zonulin is a gut barrier-regulating protein that opens tight junctions and regulates intercellular communication (7, 8). Zonulin is a precursor to haptoglobin-2 (hp-2). Haptoglobins are acute-phase reaction proteins that form complexes with hemoglobin to prevent oxidative damage (17). Following the research by Scheffler et al. and Ajamian et al., the manufacturer expressed concerns about the test’s ability to accurately detect zonulin. Ajamian et al. reported that the ELISA detects complement factor C3 and albumin (11). Scheffler et al. found discrepancies between ELISA-measured zonulin levels and Hp genotypes, suggesting that the test does not detect pure pre-haptoglobin 2 but rather structurally similar molecules, likely from the mannan-binding lectin-associated serine proteases (MASP) family. Mass spectrometry and Western blot analyses identified one of the detected molecules as properdin, a complement-associated protein with zonulin-like properties (10). Fasano, who first described zonulin, replied that zonulin represents a family of structurally and functionally related proteins involved in epithelial barrier regulation (18, 20). Therefore, the ELISA that previously measured zonulin has been renamed to measure “zonulin family peptide” (ZFP). This assumption, however, has been questioned by other researchers. The existence of such a protein family remains unconfirmed, and several of the proposed complement-associated ZFP could not be consistently detected in the liver, although the liver is assumed to be the main site of production (12). The zonulin (now referred to as zonulin family peptide, ZFP) ELISA is based on the synthetic octapeptide sequence GGVLVQPG, also known as AT1001. Initially, GGVLVQPG was thought to correspond to a fragment of fetal zonulin (8, 10). However, subsequent studies have raised questions regarding this assumption (21). Furthermore, the role of ZFP in gastrointestinal permeability remains a subject of debate (12). ZFP does not appear to be able to make a statement about gastrointestinal permeability (22). However, ZFP appears to be involved in inflammatory processes. Elevated levels of ZFP have been found in patients suffering from rheumatoid arthritis (RA) (23). Patients without RA, but with presence of elevated ZFP levels, had a higher risk of developing RA within one year. The authors suggest that the ZFP levels reflect the extent of gastrointestinal dysbiosis, which could contribute to the development of RA (23). Other researchers reported similar results in patients suffering from RA (24). Jian et al. suggest that ZFP interacts differently with bacteria compared to zonulin (12). Pietrukaniec et al. reported that ZFP levels were significantly lower in cirrhotic patients compared to healthy controls. A decrease in serum ZFP levels correlated with lower ascites ZFP levels, suggesting that reduced ZFP levels in cirrhosis reflect decreased liver synthesis rather than increased gut permeability (25). There are only a few studies that have investigated ZFP. Many recent studies have measured zonulin using ELISA, raising the question of whether ZFP might have been measured in these studies instead. Elevated levels of ZFP may be associated with fatty liver disease and metabolic syndrome, although these studies discuss zonulin, it is likely that ZFP was actually measured instead (26, 27). However, the results of our trial surprised us, as we found elevated ZFP levels in almost all patients before surgery. One possible explanation for the increase of ZFP could be the abdominal disease of our patients, although Tajik et al. reported that their cancer patients did not have elevated values (23). In our cohort, ZFP levels were also not associated with the presence of malignancy. According to Fasano, ZFP is a term that encompasses various types of acute phase proteins that can be elevated in abdominal diseases (18, 20). Other pre-existing conditions may have contributed to elevated ZFP levels. Factors such as medication use, exposure to radiation or chemotherapy, inflammatory bowel disease, malignancies, and psychological stress can all disrupt the gut microbiome, promote intestinal dysbiosis, and potentially lead to increased serum concentrations of ZFP. Nevertheless, a significant degree of uncertainty persists at this point.

To summarize, this study aimed to investigate the potential of ZFP as a biomarker for early detection of postoperative complications in colorectal surgery. While our results did not show a significant association between ZFP levels and the development of minor complications, the study offers valuable insights into the biodynamics of ZFP before and after colorectal resection. Despite challenges such as patient dropouts and variability in blood sample availability, the structured measurement of ZFP levels over multiple time points allowed us to observe trends in its postoperative fluctuations. The data suggests that ZFP does not serve as a marker for predicting postoperative minor complications. Given the current state of knowledge described above, the measurement of ZFP is associated with considerable uncertainty. It is not well understood which specific proteins or fragments are detected by the commonly used assays, and the lack of analytical specificity raises doubts about the reliability of the measured values during the last years. Furthermore, the regulation and biological variability of ZFP are still poorly understood. A range of factors may influence circulating ZFP levels, including inflammation, medication, and alterations in the gut microbiome. From a clinical perspective, we do not recommend ZFP measurement in patients following colorectal resection.

## Patient consent statement

All included patients gave their written informed consent before study participation.

## Data Availability

The raw data supporting the conclusions of this article will be made available by the authors, without undue reservation.
